# Signature based on RNA-binding protein-related genes for predicting prognosis and guiding therapy in non-small cell lung cancer

**DOI:** 10.3389/fgene.2022.930826

**Published:** 2022-09-02

**Authors:** Ti-Wei Miao, Fang-Ying Chen, Long-Yi Du, Wei Xiao, Juan-Juan Fu

**Affiliations:** ^1^ Department of Integrated Traditional Chinese and Western Medicine, West China Hospital, Sichuan University, Chengdu, China; ^2^ West China School of Medicine, West China Hospital, Sichuan University, Chengdu, China

**Keywords:** RNA-binding proteins, non-small cell lung cancer, prognosis, immune infiltration, therapy, bioinformatics

## Abstract

**Background:** Studies have reported that RNA-binding proteins (RBPs) are dysregulated in multiple cancers and are correlated with the progression and prognosis of disease. However, the functions of RBPs in non-small cell lung cancer (NSCLC) remain unclear. The present study aimed to explore the function of RBPs in NSCLC and their prognostic and therapeutic value.

**Methods:** The mRNA expression profiles, DNA methylation data, gene mutation data, copy number variation data, and corresponding clinical information on NSCLC were downloaded from The Cancer Genome Atlas, Gene Expression Omnibus, and the University of California Santa Cruz Xena databases. The differentially expressed RBPs were identified between tumor and control tissues, and the expression and prognostic value of these RBPs were systemically investigated by bioinformatics analysis. A quantitative polymerase chain reaction (qPCR) was performed to validate the dysregulated genes in the prognostic signature.

**Results:** A prognostic RBP-related signature was successfully constructed based on eight RBPs represented as a risk score using least absolute shrinkage and selection operator (LASSO) regression analysis. The high-risk group had a worse overall survival (OS) probability than the low-risk group (*p* < 0.001) with 1-, 3-, and 5-year area under the receiver operator characteristic curve values of 0.671, 0.638, and 0.637, respectively. The risk score was associated with the stage of disease (*p* < 0.05) and was an independent prognostic factor for NSCLC when adjusted for age and UICC stage (*p* < 0.001, hazard ratio (HR): 1.888). The constructed nomogram showed a good predictive value. The *P53*, focal adhesion, and NOD-like receptor signaling pathways were the primary pathways in the high-risk group (adjusted *p* value <0.05). The high-risk group was correlated with increased immune infiltration (*p* < 0.05), upregulated relative expression levels of programmed cell death 1 (*PD1*) (*p* = 0.015), cytotoxic T-lymphocyte-associated protein 4 (*CTLA4*) (*p* = 0.042), higher gene mutation frequency, higher tumor mutational burden (*p* = 0.034), and better chemotherapy response (*p* < 0.001). The signature was successfully validated using the GSE26939, GSE31210, GSE30219, and GSE157009 datasets. Dysregulation of these genes in patients with NSCLC was confirmed using the qPCR in an independent cohort (*p* < 0.05).

**Conclusion:** An RBP-related signature was successfully constructed to predict prognosis in NSCLC, functioning as a reference for individualized therapy, including immunotherapy and chemotherapy.

## Introduction

Non-small cell lung cancer (NSCLC) is the leading cause of cancer-related death worldwide with an incidence rate of 1.3 million cases per year (Bray et al., 2018). NSCLC remains asymptomatic during the early stage, and only 25% of patients with NSCLC are diagnosed at this stage (Ni et al., 2018). However, approximately 80% of patients are diagnosed in metastatic stages with a 5-year survival rate below 15% (Goldstraw et al., 2016). Despite constant progress in novel therapies for NSCLC, including targeted therapy, immunotherapy, and chemotherapy, the therapeutic efficacy is still unsatisfactory (Facchinetti et al., 2016). Thus, the identification of effective biomarkers to accurately predict the overall survival (OS) probability and guide therapy in NSCLC is of great importance.

RNA-binding proteins (RBPs) are a group of proteins that function with an RNA-binding domain to distinguish and bind to target RNAs, including coding RNAs and non-coding RNAs (Hong, 2017). To date, more than 1,500 RBPs have been identified in the human genome through high-throughput screening (Cook et al., 2011; Gerstberger et al., 2014). RBPs modulate the fate of binding RNAs by regulating transcription, editing, splicing, polyadenylation, translocation, and turnover (Müller-McNicoll and Neugebauer, 2013). In recent years, studies in genetics and proteomics have shown that most RBPs exhibit functional abnormalities in lung cancer. Quaking I-5 (*QKI-5*), RALY heterogeneous nuclear ribonucleoprotein (*RALY*), and KH-type splicing regulatory protein (*KHSRP*) promote cancer cell proliferation and invasion, and they are associated with OS probability in NSCLC (Yan et al., 2019; Liang et al., 2020a; Song et al., 2020). Musashi1 (*MSI1*) promotes NSCLC malignancy and chemoresistance (Lang et al., 2017). RNA-binding motif protein 47 (*RBM47*) inhibits NSCLC metastasis through modulation of AXIN1 mRNA stability and Wnt/β-catenin signaling (Shen et al., 2020). However, the molecular mechanism of RBPs and their prognostic predictive ability in NSCLC remain unknown. A systematic analysis of RBPs has reported novel and comprehensive insights into the underlying mechanism during cancer progression, and prognostic signatures using RBPs have been constructed in multiple tumors (Li et al., 2020a; Li et al., 2020b; Li et al., 2020c; Kang et al., 2020; Wang et al., 2020). However, a prognostic RBP-related signature in NSCLC has not been reported. Thus, the present study aimed to explore the function of RBPs in NSCLC and their prognostic and therapeutic value.

In the present study, mRNA expression profiles, DNA methylation data, gene mutation data, copy number variation data, and clinical information on NSCLC were obtained from The Cancer Genome Atlas (TCGA, https://portal.gdc.cancer.gov/repository), Gene Expression Omnibus (GEO, https://www.ncbi.nlm.nih.gov/geo/), and the University of California Santa Cruz (UCSC) Xena (https://xenabrowser.net/) databases. The differentially expressed RBPs (DERBPs) were screened and then applied to perform functional enrichment analysis and to construct a prognostic RBP-related signature. A nomogram was created, and Kaplan–Meier survival analysis and Cox regression analysis were performed to explore its prognostic value. The underlying molecular mechanisms between the different risk groups were investigated using gene set enrichment analysis (GSEA). The correlations of risk score with clinical characteristics, DNA methylation levels, tumor mutational burden (TMB), immune infiltration, and chemotherapy sensitivity were analyzed using R software packages. Finally, dysregulated expression levels of these genes were validated using the quantitative polymerase chain reaction (qPCR).

## Materials and methods

### Data preprocessing and identification of DERBPs

The following data were downloaded from TCGA database: mRNA expression profiles of 1,037 NSCLC samples and 108 control samples; DNA methylation data on 807 NSCLC samples and 71 control samples; gene mutation data on 1,059 NSCLC samples; and clinical information (age, gender, smoking history, Union for International Cancer Control (UICC) stage, survival time, and survival status) on 1,027 tumor samples. Moreover, the GSE31210, GSE26939, GSE30219, and GSE157009 datasets were obtained from the GEO database. The GSE31210, GSE30219, and GSE157009 datasets were generated using the GPL570 [HG-U133_Plus_2] Affymetrix Human Genome U133 Plus 2.0 Array, whereas the GSE26939 dataset was generated using the GPL9053 Agilent-UNC-custom-4X44K. The GSE31210 dataset included mRNA expression profiles of 226 NSCLC samples and 20 control samples, as well as clinical information on 226 NSCLC samples. The GSE26939 dataset included mRNA expression profiles of 116 NSCLC samples and 0 control samples, as well as clinical information on 116 NSCLC samples. The GSE30219 dataset included mRNA expression profiles of 293 NSCLC samples and 14 control samples, as well as clinical information on 293 NSCLC samples. The GSE157009 dataset included mRNA expression profiles of 249 NSCLC samples and 0 control samples, as well as clinical information on 249 NSCLC samples. Copy number variation data on 1079 NSCLC samples were downloaded from the UCSC Xena database. The inclusion criteria were as follows: 1) NSCLC; 2) mRNA expression profiles, and 3) complete follow-up data. Therefore, 978 patients from TCGA, 226 patients from the GSE31210 dataset, 114 patients from the GSE26939 dataset, 264 patients from the GSE30219 dataset, and 248 patients from the GSE157009 dataset were enrolled in the present study. The baseline characteristics of the patients are shown in Table 1. A total of 1,542 RBPs were included in the present study (Cook et al., 2011; Gerstberger et al., 2014). DERBPs were identified based on |log_2_ fold change (FC)| ≥ 0.7 and adjusted *p* value <0.05 using the “limma” package in R (Version 4.0.2) when two groups were compared. The “ggplot2” and “gplots” packages in R were used to generate the volcano plots and heatmap.

**TABLE 1:** Clinical features of patients with NSCLC from TCGA and GEO databases.

Clinical characteristic	TCGA cohort (978)	GSE31210 (226)	GSE26939 (114)	GSE30219 (264)	GSE157009 (248)
Age (years)					
≥65	590 (60.33%)	62 (0.27%)	61 (53.51%)	104 (39.39%)	182 (73.39%)
<65	388 (39.67%)	164 (72.57%)	53(46.49%)	159 (60.23%)	66 (26.61%)
Unknown	0	0	0	1 (0.38%)	0
Gender					
Male	585 (59.82%)	105 (46.46%)	52 (46.61%)	223 (84.47%)	160 (64.52%)
Female	393 (40.185)	121 (53.54)	62 (54.39%)	41 (15.53%)	88 (35.48%)
Unknown	0	0	0	0	0
T classification					
T1–T2	819 (83.74%)	226 (100%)	—	218 (82.58%)	230 (92.74%)
T3–T4	156 (15.95%)	0	—	44 (16.67%)	18 (7.26%)
Unknown	3 (0.31%)	0	—	2 (0.76%)	0
N classification					
N0	629 (64.31%)	—	—	188 (71.21%)	—
N1–N3	333 (34.05%)	—	—	74 (28.03)	—
Unknown	16 (1.64%)	—	—	2 (0.76%)	—
M classification					
M0	722 (73.82%)	—	—	257 (97.35%)	—
M1	31 (3.17%)	—	—	4 (1.52)	—
Unknown	225 (23.01%)	—	—	3 (1.14%)	—
UICC stage					
Stage I–II	772 (78.94%)	226 (100%)	81 (71.05%)	208 (78.79%)	—
Stage III–IV	194 (19.84%)	0	20 (17.54%)	52 (19.70%)	—
Unknown	12 (1.23%)	0	13 (11.40%)	4 (1.52%)	—

TCGA, The Cancer Genome Atlas; GEO, Gene Expression Omnibus; NSCLC, non-small cell lung cancer; UICC, Union for International Cancer Control.

### Functional enrichment analyses

Functional enrichment analyses of the DERBPs were performed by Gene Ontology (GO) and Kyoto Encyclopedia of Genes and Genomes (KEGG) pathway analyses using the “clusterProfiler,” “org.Hs.eg.db,” “enrichplot,” “ggplot2,” and “GOplot” packages in R. GO analysis included biological processes (BPs), cellular components (CCs), and molecular functions (MFs). The adjusted *p* value <0.05 was considered statistically different.

### Construction and validation of the prognostic RBP-related signature

The prognostic RBPs were screened using DERBPs through univariate Cox regression analysis, and *p* < 0.05 was selected as the statistical threshold. The least absolute shrinkage and selection operator (LASSO) regression analysis was applied to identify hub DERBPs to minimize the risk of overfitting among the signatures. The changing trajectory of each independent variable was first analyzed, and fivefold cross-validation was used to build a model and analyze the confidence interval under each lambda value. The following formula was utilized: risk score = expression for each gene x coefficient for each gene. The patients were divided into low- and high-risk groups, according to the median risk score. A Kaplan–Meier survival curve was constructed between the two risk groups and was compared using the log-rank test. A receiver operating characteristic (ROC) curve was used to assess the predictive value of the Kaplan–Meier survival curves, and 1-, 3-, and 5-year area under the curve (AUC) values were calculated. The prognostic signature was validated using the following independent cohorts: GSE31210, GSE26939, GSE30219, and GSE157009.

### Correlation of the risk score with clinical characteristics

The risk score was compared in different age groups (≥65 and <65 years), genders (female and male), UICC stages (stage I–II and stage III–IV), T stages (T1–2 and T3–4), N stages (N0 and N1–3), and M stages (M0 and M1) by the Mann–Whitney test in TCGA cohort. Univariate and multivariable Cox regression analyses were performed to screen independent prognostic factors for NSCLC in TCGA, GSE31210, and GSE26939 datasets by R software.

### Nomogram and calibration plots

A nomogram was applied to forecast the likelihood of OS probability using independent prognostic factors for NSCLC through the “rms” package in R. Calibration plots of the nomogram were generated to evaluate the conformity of the nomogram predicted and actual OS probability.

### Gene set enrichment analysis

GSEA is a computational method that determines whether a previously defined set of genes shows a significant difference between two biological states (Subramanian et al., 2005). The “c2. cp.kegg.v7.5.1. symbols.gmt” file was downloaded from the GSEA database (http://www.gsea-msigdb.org/gsea/index.jsp). The “limma,” “GSEABase,” “GSVA,” and “pheatmap” packages in R were applied to perform KEGG pathway analysis between the two risk groups. The adjusted *p* value <0.05 was considered statistically significant.

### DNA methylation, TMB, and copy number variation analyses

DNA methylation data on NSCLC were obtained by Strawberry Perl (5.32.1.1–64-bit). DNA methylation levels of prognostic genes were extracted using the “limma” package in R and compared between the two risk groups by the Mann–Whitney test. Gene mutation data on lung adenocarcinoma (LUAD) and lung squamous cell carcinoma (LUSC) were allocated in different “maf” files in TCGA database, and the gene mutation frequencies in the two risk groups in LUAD and LUSC were evaluated using the “maftools” package in R. The correlation of risk score with TMB was evaluated by the Mann–Whitney test and Spearman’s rank correlation analysis. Kaplan–Meier survival curve analysis was performed on the high-risk score + high TMB group and the low-risk score + low TMB group, and the curves were compared by the log-rank test by R software. Copy number variation matrixes of NSCLC were obtained by Strawberry Perl. The copy number variation levels and positions of prognostic genes were analyzed using the “RCircos” package in R.

### Immune infiltration analysis

The mRNA expression matrix of NSCLC was converted into a tumor microenvironment (TME) score matrix using the “limma” and “estimate” packages in R. TME scores, including immune score, stromal score, and estimate score, were compared between the two risk groups using the “reshape2” and “ggpubr” packages in R. Immune infiltration profiles were compared between the two risk groups and visualized by a violin plot using the “vioplot” package in R. The correlations of immune cells with risk scores were evaluated using the “limma,” “reshape2,” “tidyverse,” “ggplot2,” “ggpubr,” and “ggExtra” packages in R. Comparisons were performed by Spearman’s rank correlation analysis based on *p*-value <0.05 and |r| > 0.1. Relative expression levels of immune checkpoint inhibitors (programmed cell death 1 (*PD1*), programmed cell death ligand 1 (*PDL1*), and cytotoxic T-lymphocyte-associated protein 4 (*CTLA4*)) were compared between the two risk groups.

### Sensitivity of chemotherapy drugs

Nine chemotherapy drugs, namely, axitinib (Solomon et al., 2022), dasatinib (Kim et al., 2021), docetaxel (Xiao et al., 2022), erlotinib (Wang et al., 2022), gemcitabine (Guo et al., 2022), metformin (Arrieta et al., 2022), paclitaxel (Saito et al., 2022), parthenolide (Li et al., 2020d; Sun et al., 2020), and shikonin (Pan et al., 2021), were screened from the previous literature that demonstrated their antitumor efforts on lung cancer, and they were selected for the present study. The half inhibitory concentration (IC50) of the chemotherapy drugs was compared between the two risk groups in NSCLC using the “pRRophetic” package in R. A *p*-value < 0.05 was considered statistically significant.

### RNA extraction and qPCR validation

Lung tissue samples from patients with NSCLC were obtained from the West China Hospital of Sichuan University. Histologically normal tissues were used as controls. Total RNA was extracted from the lung tissues (14 control and 13 NSCLC samples) using the E. Z.N.A. HP Total RNA Kit (OMEGA, United States), according to the manufacturer’s protocol. Complementary DNA (cDNA) was synthesized using the PrimeScript™ RT reagent Kit (TaKaRa, Japan), following the manufacturer’s instructions. Quantitative PCR was performed in triplicate using the Iq™ SYBR Green SuperMix (BIO-RAD, United States), according to the manufacturer’s protocol. The relative gene expression levels were normalized *via* the *β-actin* Ct value, applying the 2^−ΔΔCt^ relative quantification method. The following qPCR primers were used:

*ZC3H12C*‐forward, 5′‐GGC​TTT​TGA​GTC​GGA​CGG​TA‐3′; *ZC3H12C*‐reverse, 5′‐TCA​GGG​GGC​ATG​AAC​TTG​TC‐3′; *SM AD9*‐forward, 5′‐GTT​TGT​TAC​GAG​GAG​CCC​CA‐3′; *SMAD9*‐reverse, 5′‐AGG​GTC​GGT​GAA​CCC​ATC​TA‐3′; *MRPL15*‐forwa rd, 5′‐GAG​AGG​TGT​GAC​CAT​CCA​GC‐3′; *MRPL15*‐reverse, 5′‐TTG​GAA​TGG​GTT​GTC​CAC​GAA‐3′; *MBNL2*‐forward, 5′‐ATA​CGG​CAG​ACG​GCT​TTC​AG‐3′; *MBNL2*‐reverse, 5′‐CTC​TGC​CTG​TCC​TTC​CCA​TT‐3′; *FASTKD3*‐forward, 5′‐GAT​GGA​AAC​CCT​GCC​TGA​CA‐3′; *FASTKD3*‐reverse, 5′‐CCA​GGT​TCA​GCA​ACA​GGC​TA‐3′; *SNRPB*‐forward, 5′‐AAG​GGA​AGA​GAA​GCG​AGT​CC‐3′; *SNRPB*‐reverse, 5′‐GCA​AGT​GGA​ACT​CGA​GCA​AT‐3′;

*IGF2BP1*‐forward: 5′‐TAG​CTC​CTT​TAT​GCA​GGC​TCC‐3′; *IGF2BP1*‐reverse, 5′‐CGG​GAG​AGC​TGT​TTG​ATG​TG‐3′; *INT S7*‐forward, 5′‐CAC​TAT​CAG​GGA​CCA​TCG​CC‐3′;

*INTS7*‐reverse, 5′‐GGT​AAC​AGC​ACT​CTT​GGG​CT‐3′; *β-actin*‐forward, 5′‐CCA​CGA​AAC​TAC​CTT​CAA​CTC​C‐3′; *β-actin* ‐reverse, 5′‐GTG​ATC​TCC​TTC​TGC​ATC​CTG​T‐3′.

### Statistical analysis

Statistical analysis was performed by R (Version 4.0.2) and GraphPad Prism (Version 7.00) software. Levels of mRNA expression were expressed as the median (interquartile range), according to the data distribution type. Comparisons between the two groups were determined by the Mann–Whitney test for nonparametric data. Survival curves were compared by the log-rank test. Correlation analysis was performed by Spearman’s rank correlation analysis. *p* < 0.05 was considered statistically significant.

## Results

### Identification of DERBPs and functional enrichment analysis

**FIGURE 1:**
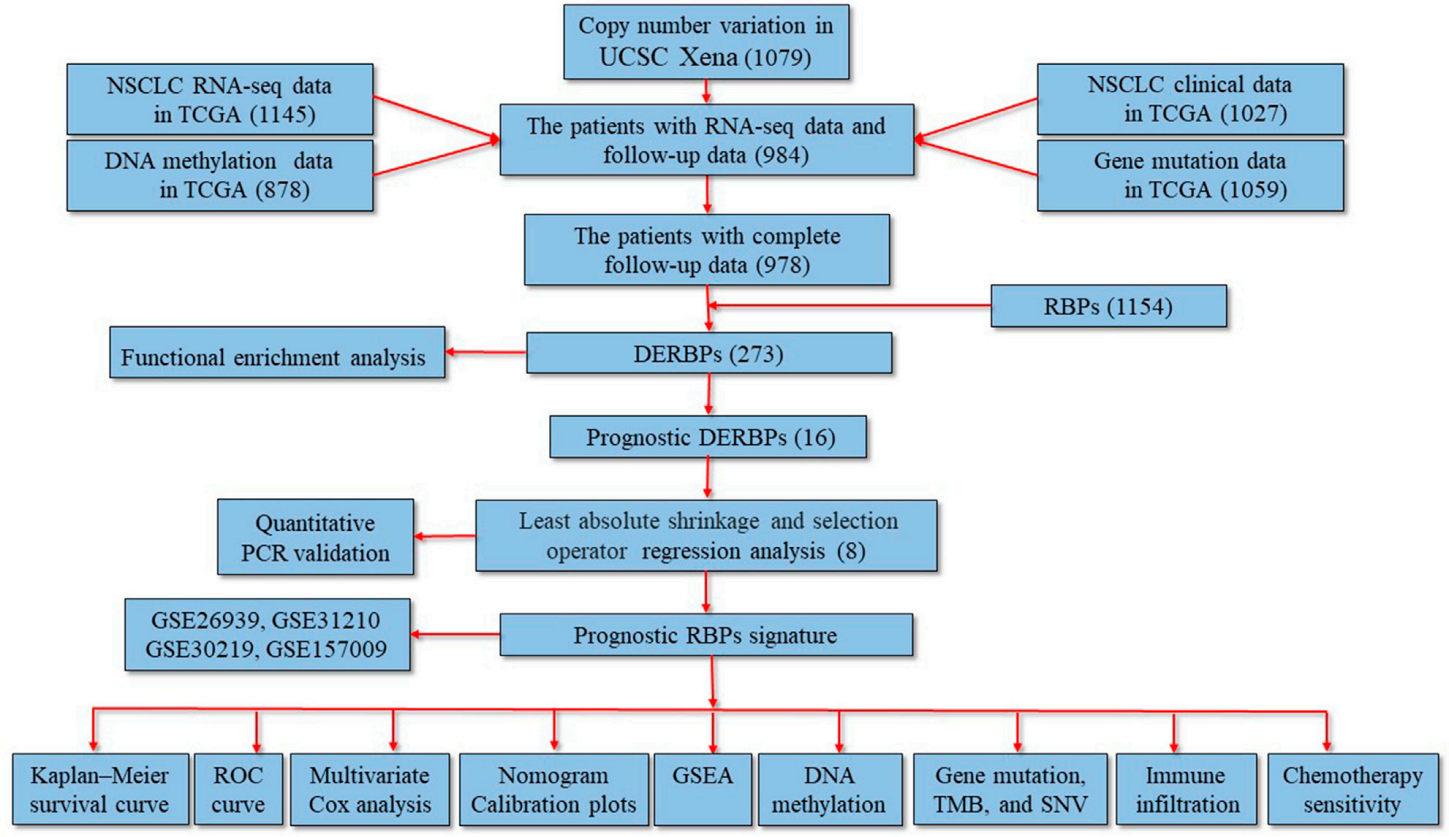
Study flowchart and the main findings of the study. The numbers within the parentheses indicate the size of the sample obtained. DERBPs, differentially expressed RNA-binding proteins; GSEA, gene set enrichment analysis; NSCLC, non-small cell lung cancer; PCR: polymerase chain reaction; ROC, receiver operating characteristic; TCGA, The Cancer Genome Atlas; TMB, tumor mutational burden; UCSC, University of California Santa Cruz.

**FIGURE 2:**
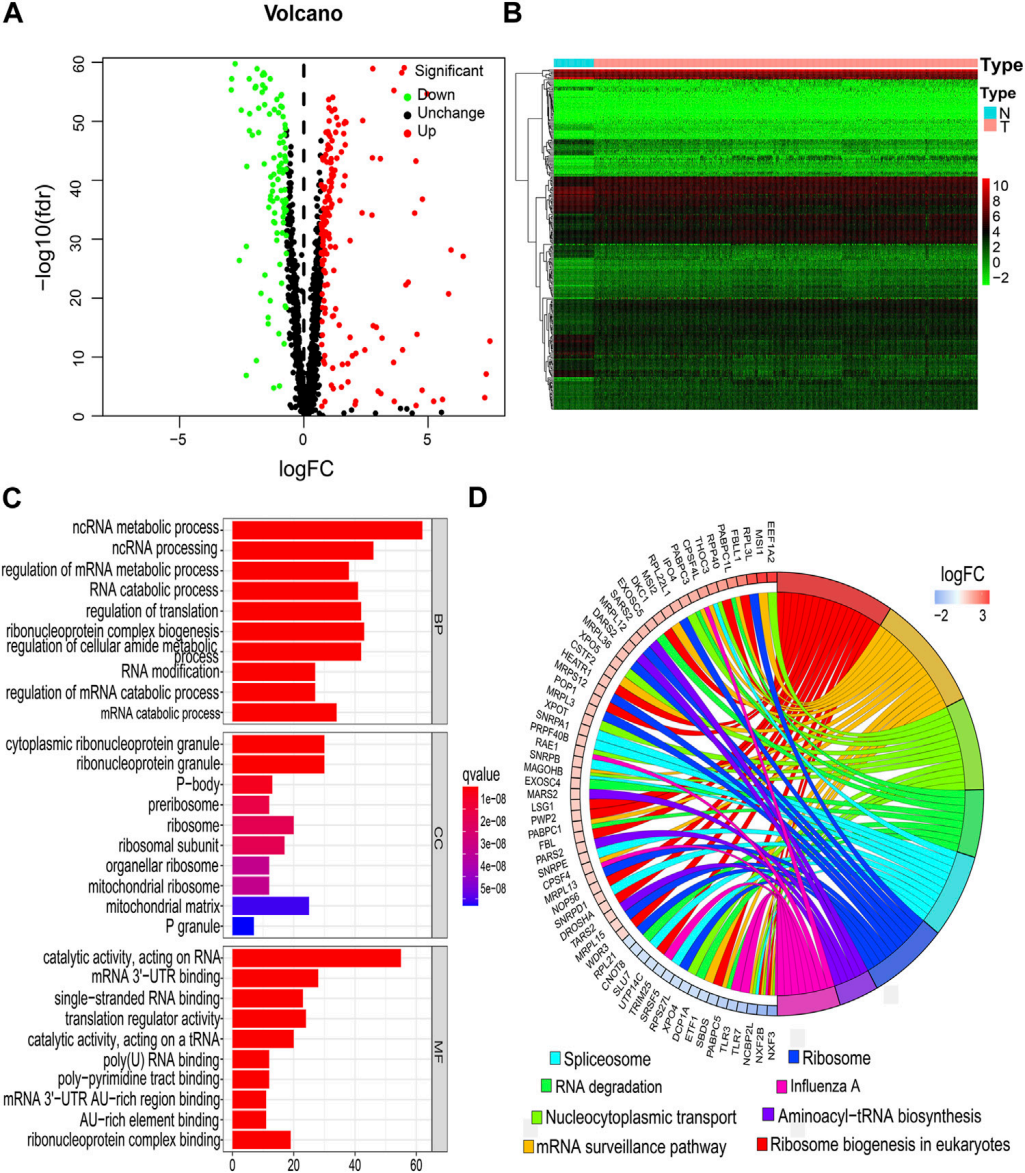
Identification of DERBPs and functional enrichment analysis. **(A)** Volcano plots. **(B)** Heatmap. **(C)** BPs, CCs, and MFs. **(D)** KEGG pathways. BPs, biological processes; CCs, cellular components; DERBPs, differentially expressed RNA-binding proteins; FC, fold-change; FDR, false discovery rate; KEGG, Kyoto Encyclopedia of Genes and Genomes; MFs, molecular functions; N, normal samples; T, tumor samples.

The study flowchart is shown in Figure 1. A total of 273 DERBPs (173 upregulated RBPs and 100 downregulated RBPs) were identified when NSCLC samples were compared to control samples (Figures 2A,B). GO analysis using 273 DERBPs identified the following enriched terms: BPs, including the ncRNA metabolic process, ncRNA processing, and regulation of the mRNA metabolic process (adjusted *p* value <0.05, Figure 2C); CCs, including those located in cytoplasmic ribonucleoprotein granules, ribonucleoprotein granules, and ribosomes (adjusted *p* value <0.05, Figure 2C); and MFs, including the catalytic activity, acting on RNA, mRNA 3'−UTR binding, and single−stranded RNA binding (adjusted *p* value <0.05, Figure 2C). KEGG pathway analysis identified ribosome biogenesis in eukaryotes, the mRNA surveillance pathway, and influenza A as the primary pathways in NSCLC (adjusted *p* value <0.05, Figure 2D).

### Construction and validation of the prognostic RBP-related signature

**FIGURE 3:**
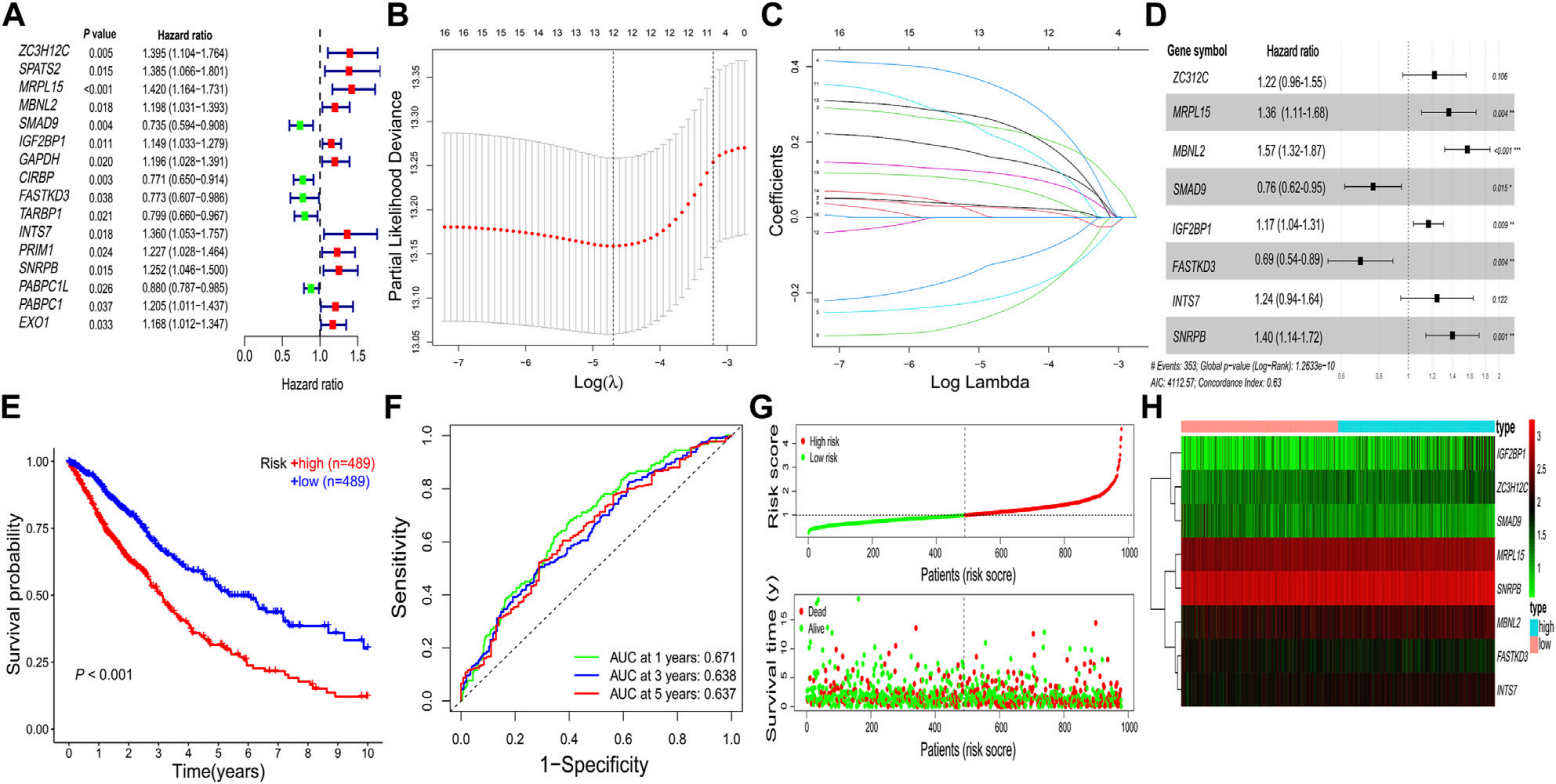
Construction of the prognostic RBP-related signature. **(A)** Univariate Cox regression analysis. **(B–D)** LASSO regression analysis. **(E)** Kaplan–Meier survival curve. **(F)** Receiver operating characteristic curve. **(G)** Risk score distribution and survival status. **(H)** Heatmap of prognostic genes. AUC, area under the curve; LASSO, least absolute shrinkage and selection operator; RBPs, RNA-binding proteins; *p < 0.05; **p < 0.01; ***p < 0.001.

A total of 16 prognostic RBPs were screened from 273 DERBPs using univariate Cox regression analysis (*p* < 0.05, Figure 3A). An eight-RBP signature (zinc finger CCCH-type containing 12C (*ZC3H12C*), mitochondrial ribosomal protein L15 (*MRPL15*), muscleblind-like splicing regulator 2 (*MBNL2*), SMAD family member 9 (*SMAD9*), insulin-like growth factor 2 mRNA binding protein 1 (*IGF2BP1*), FAST kinase domains 3 (*FASTKD3*), integrator complex subunit 7 (*INTS7*), and small nuclear ribonucleoprotein polypeptides B and B1 (*SNRPB*)) were generated utilizing LASSO regression analysis (Figures 3B–D). The formula for calculating the risk score was as follows: Risk score = (0.20 * *ZC3H12C*exp) + (0.31 * *MRPL15*exp) + (0.45 * *MBNL2*exp) + (-0.27 * *SMAD9*exp) + (0.15 * *IGF2BP1*exp) + (-0.37 * *FASTKD3*exp) + (0.22 * *INTS7*exp) + (0.34 * *SNRPB*exp).

**FIGURE 4:**
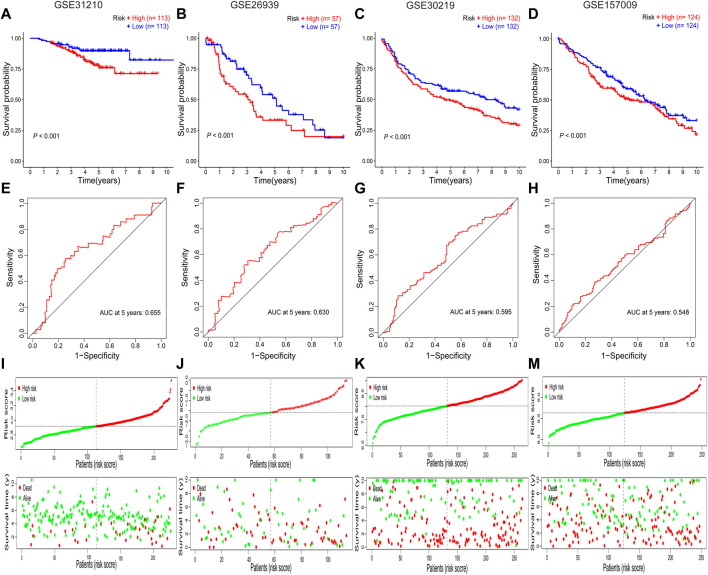
Validation of the prognostic RBP-related signature. **(A–D)** Kaplan–Meier survival curves for the GSE31210, GSE26939, GSE30219, and GSE157009 datasets. **(E–H)** Receiver operating characteristic curves for the GSE31210, GSE26939, GSE30219, and GSE157009 datasets. **(I–M)** Risk score distribution and survival status for the GSE31210, GSE26939, GSE30219, and GSE157009 datasets. AUC, area under the curve; RBPs: RNA-binding proteins.

All patients with NSCLC were divided into low-or high-risk groups, according to the median risk score of 0.9867 (Figure 3G). The high-risk group had a worse OS probability than the low-risk group (*p* < 0.001; Figure 3E), and the 1-, 3-, and 5-year AUC values were 0.671, 0.638, and 0.637, respectively (Figure 3F). The high-risk group had higher mortality than the low-risk group (Figure 3G). A heatmap was generated to show the different expression profiles of the eight RBPs between the two risk groups (Figure 3H). Kaplan–Meier survival curve analysis showed that the OS probability was significantly worse in the high-risk group than in the low-risk group in the GSE31210 (*p* < 0.001; Figure 4A), GSE26939 (*p* < 0.001; Figure 4B), GSE30219 (*p* < 0.001; Figure 4C), and GSE157009 (*p* < 0.001; Figure 4D) datasets. The 5-year AUC value was 0.655 in the GSE31210 dataset (Figure 4E), 0.630 in the GSE26939 dataset (Figure 4F), 0.595 in the GSE30219 dataset (Figure 4G), and 0.548 in the GSE157009 dataset (Figure 4H). The risk score and survival status in the GSE31210, GSE26939, GSE30219, and GSE157009 datasets are shown in Figures 4I–K and 4M, respectively.

### Association of risk scores with clinical characteristics

**FIGURE 5:**
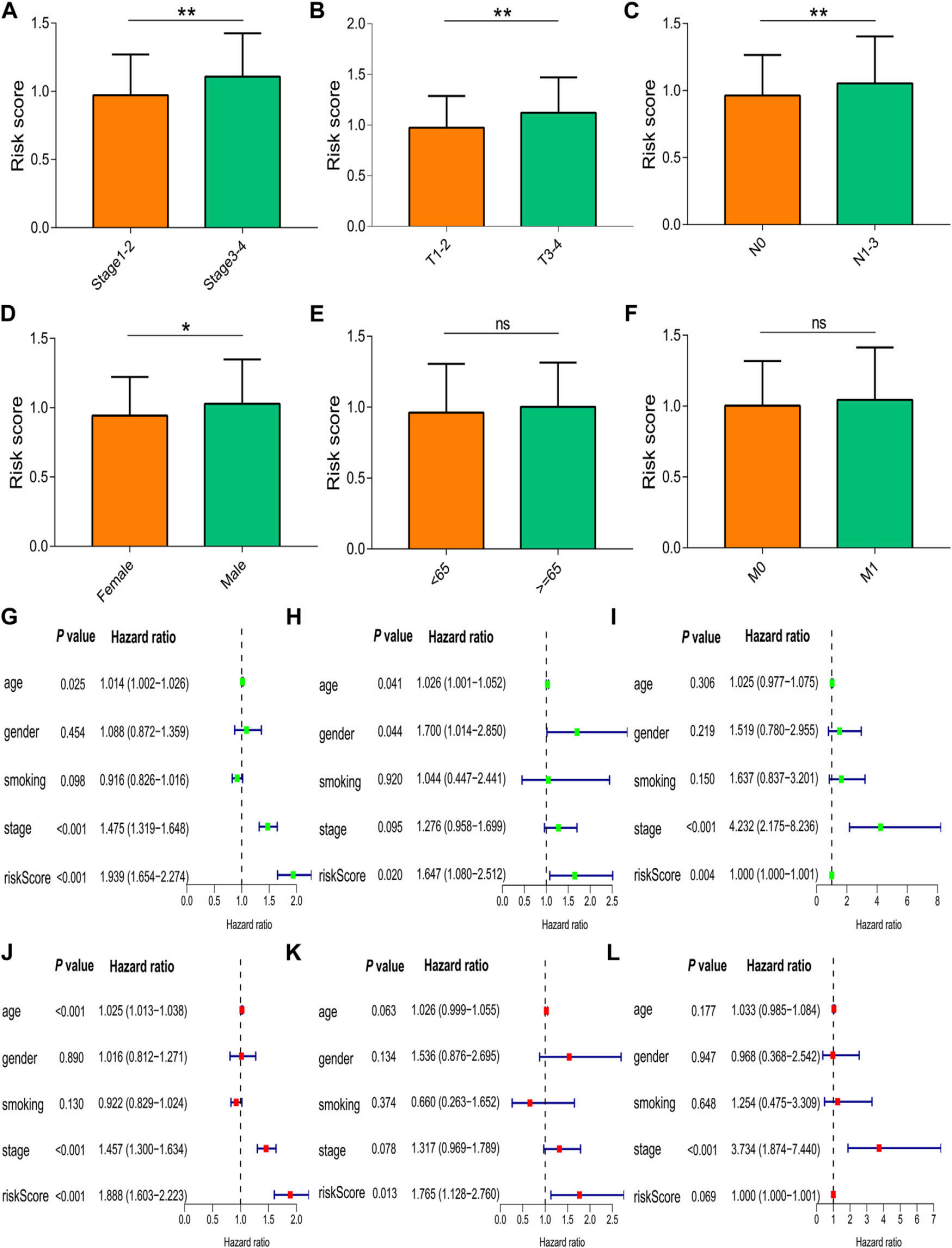
Correlation of the risk score with clinical characteristics. **(A)** UICC stages. **(B)** T stages. **(C)** N stages. **(D)** Gender groups. **(E)** Age groups. **(F)** M stages. Univariate and multivariate Cox regression analyses in **(G,J)** TCGA, **(H,K)** GSE26939, and **(I,L)** GSE31210. Data are presented as the median (interquartile range). TCGA, The Cancer Genome Atlas; UICC, Union for International Cancer Control; ns, no significance; *p < 0.05; **p < 0.01; ***p < 0.001.

The risk score in patients with stage III-IV cancer was increased compared to that in patients with stage I-II cancer (*p* = 0.004, Figure 5A). The risk score was higher in patients with T3-4 cancer than in patients with T1-2 cancer (*p* = 0.003, Figure 5B), and the risk score was increased in patients with N1–3 cancer compared to patients with N0 cancer (*p* = 0.009, Figure 5C). The risk score of male patients was higher than that of female patients (*p* = 0.020, Figure 5D), and the risk score did not differ in different age groups (*p* = 0.340, Figure 5E) or M stages (*p* = 0.921, Figure 5F). Univariate Cox regression analysis showed that age, UICC stage, and risk score were correlated with prognosis of NSCLC in TCGA database (hazard ratio [HR]: 1.014, *p* = 0.025; HR: 1.475, *p* < 0.001; and HR: 1.939, *p* < 0.001, Figure 5G), which was confirmed by multivariate Cox regression analysis (for age: HR: 1.025, *p* < 0.001; for UICC stage: HR: 1.457, *p* < 0.001; and for risk score: HR: 1.888, *p* < 0.001, Figure 5J). The risk score was correlated with prognosis in the GSE26939 dataset by univariate Cox regression analysis (HR: 1.647, *p* = 0.020, Figure 5H) and multivariate Cox regression analysis (HR: 1.765, *p* = 0.013, Figure 5K). The UICC stage and risk score were correlated with the prognosis of NSCLC in the GSE31210 dataset by univariate Cox regression analysis (HR: 4.232, *p* < 0.001; HR: 1.000, *p* = 0.004, Figure 5I) and multivariate Cox regression analysis (HR: 3.734, *p* < 0.001; HR: 1.000, *p* = 0.069, Figure 5L).

### Nomogram and calibration plots

**FIGURE 6:**
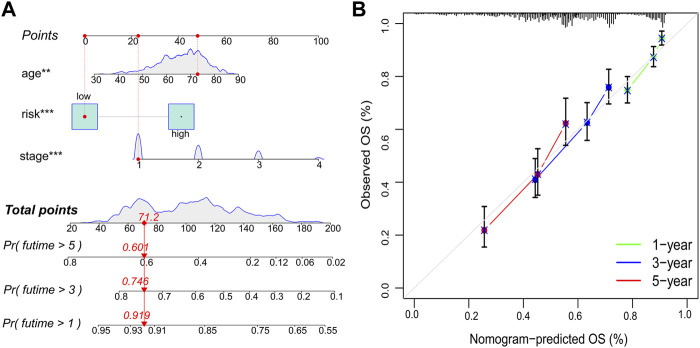
Nomogram and calibration plots. **(A)** Nomogram to predict OS probability at 1, 3, and 5 years. **(B)** Calibration plots of the nomogram. OS, overall survival; **p < 0.01; ***p < 0.001.

A nomogram was constructed using the independent prognostic factors (age, UICC stage, and risk score) to predict OS probability after 1, 3, and 5 years, which was calculated by plotting a vertical line between the total point axis and each prognostic axis (Figure 6A). Calibration plots of the nomogram showed high conformity of the nomogram predicted and actual OS probability at 1, 3, and 5 years (Figure 6B).

### GSEA

**FIGURE 7:**
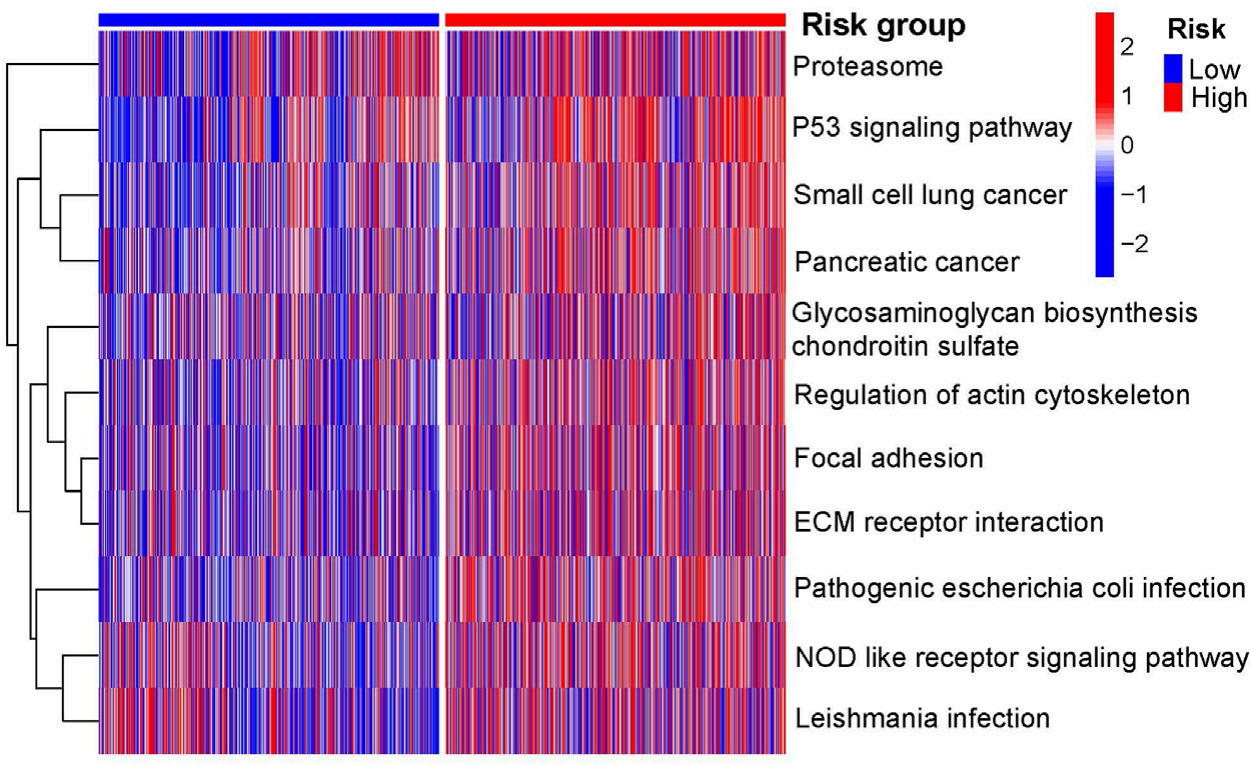
Primary KEGG pathways between the two risk groups according to GSEA. GSEA, gene set enrichment analysis; KEGG, Kyoto Encyclopedia of Genes and Genomes.

The results of the GSEA showed that the *P53*, focal adhesion, and NOD-like receptor signaling pathways were the primary enriched pathways in the high-risk group of patients with NSCLC (adjusted *p*-value <0.05, Figure 7).

### DNA methylation, TMB, and copy number variation analyses

**FIGURE 8:**
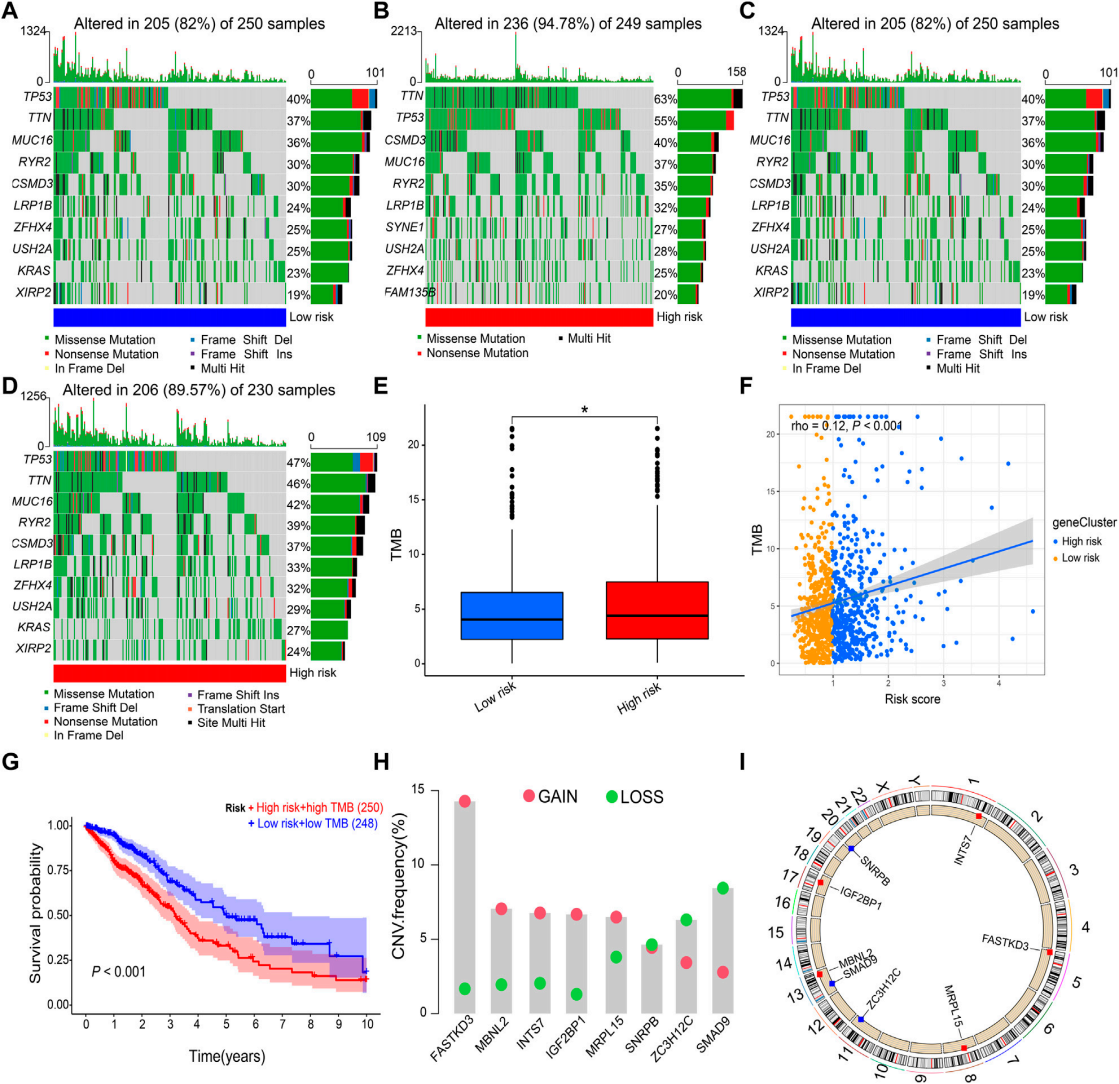
Gene mutation and copy number variation. **(A,B)** Gene mutation frequencies in the low-risk and high-risk groups of LUAD. **(C,D)** Gene mutation frequencies in the low-risk and high-risk groups of LUSC. **(E,F)** Correlation of the risk score with TMB. **(G)** Kaplan–Meier survival curve between the high-risk score + high TMB group and the low-risk score + low TMB group. **(H)** Copy number variations of prognostic genes. **(I)** Positions of prognostic genes in the chromosome. LUAD, lung adenocarcinoma; LUSC, lung squamous cell carcinoma; TMB, tumor mutational burden. Data are presented as the median (interquartile range). *p < 0.05.

The DNA methylation levels of the eight prognostic genes were not significantly different between the two risk groups (Supplementary Figure S1). Mutation analysis revealed that *TP53* and *TTN* were the most frequently mutated genes in both risk groups of LUAD (Figures 8A,B) and LUSC (Figures 8C,D). The high-risk group had a higher TMB than the low-risk group in NSCLC (*p* = 0.034, Figure 8E), and Spearman’s rank correlation analysis showed the positive correlation of TMB with risk score (rho = 0.12 and *p* < 0.001; Figure 8F). The Kaplan–Meier survival curve showed that the OS probability was lower in the high-risk score + high TMB group than in the low-risk score + low TMB group (*p* < 0.001, Figure 8G). Copy number variations were significantly increased in *FASTKD3*, *MBNL2*, *INTS7*, *IGF2BP1*, and *MRPL15*, and they were significantly decreased in *NRPB*, *ZC3H12C*, and *SMAD9* (Figure 8H). The positions of the prognostic genes in the chromosome are illustrated in Figure 8I.

### Immune infiltration analysis

**FIGURE 9:**
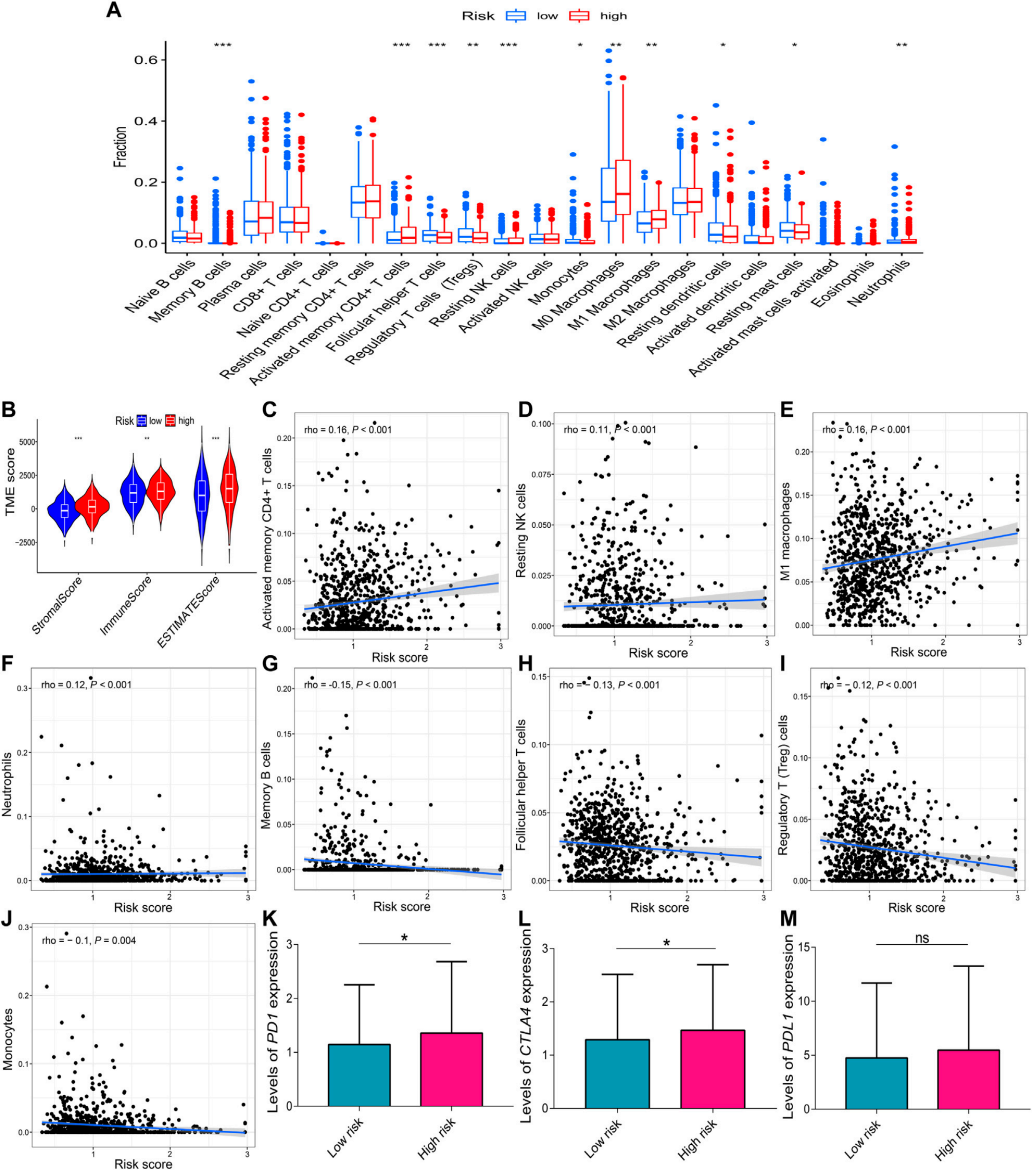
Correlations of the risk score with immune infiltration. **(A)** Comparison of the different immune infiltration profiles between the two risk groups. **(B)** TME scores of the two risk groups. A positive correlation with the risk score was observed for **(C)** activated memory CD4^+^ T cells, **(D)** resting NK cells, **(E)** M_1_ macrophages, and **(F)** neutrophils. A negative correlation with the risk score was observed for **(G)** memory B cells, **(H)** follicular helper T cells, **(I)** Treg cells, and **(J)** monocytes. **(K–M)** Relative expression levels of *PD1*, *CTLA4*, and *PDL1* in the two risk groups. Data are presented as the median (interquartile range). *CTLA4*, cytotoxic T-lymphocyte-associated protein 4; NK, natural killer; *PD1*, programmed cell death one; *PDL1*, programmed cell death one ligand 1; TME, tumor microenvironment; ns, no significance; *p < 0.05; **p < 0.01; ***p < 0.001.

Because the stromal score (*p* < 0.001), immune score (*p* < 0.01), and estimate score (*p* < 0.001) were all increased in the high-risk group compared to the low-risk group (Figure 9B), the correlations of immune cell with risk score were evaluated. The boxplot showed that the high-risk group had increased activated memory CD4^+^ T cells (*p* < 0.001), resting natural killer (NK) cells (*p* < 0.001), M_0_ and M_1_ macrophages (*p* < 0.01), neutrophils (*p* < 0.01), reduced memory B cells (*p* < 0.001), follicular helper T cells (*p* < 0.001), regulatory T (Treg) cells (*p* < 0.01), monocytes (*p* < 0.05), resting dendritic cells (*p* < 0.05), and resting mast cells (*p* < 0.05) compared to the low-risk group (Figure 9A). The risk score was positively correlated with activated memory CD4^+^ T cells (rho = 0.16; *p* < 0.001), resting NK cells (rho = 0.11; *p* < 0.001), M_1_ macrophages (rho = 0.16; *p* < 0.01), and neutrophils (rho = 0.12; *p* < 0.01) (Figures 9C–F), and it was negatively correlated with memory B cells (rho = –0.15; *p* < 0.001), follicular helper T cells (rho = –0.13; *p* < 0.001), Treg cells (rho = –0.12; *p* < 0.01), and monocytes (rho = –0.1; *p =* 0.004) (Figures 9G–J). In addition, the relative expression levels of *PD1* (*p =* 0.015) and *CTLA4* (*p =* 0.042) were higher in the high-risk group than the low-risk group (Figures 9K,L). However, the relative expression levels of *PDL1* were not significantly different between the two risk groups (*p =* 0.087, Figure 9M).

### Sensitivity of chemotherapy drugs

**FIGURE 10:**
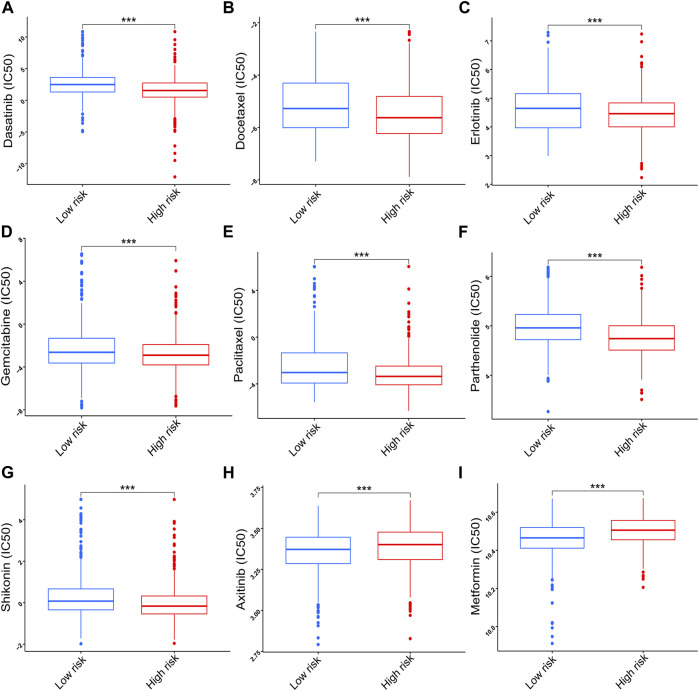
Correlation of the risk score with chemotherapy sensitivity. **(A)** Dasatinib. **(B)** Docetaxel. **(C)** Erlotinib. **(D)** Gemcitabine. **(E)** Paclitaxel. **(F)** Parthenolide. **(G)** Shikonin. **(H)** Axitinib. **(I)** Metformin. Data are presented as the median (interquartile range). IC50, half inhibitory concentration; ***p < 0.001.

To further explore the clinical value of the prognostic signature, the sensitivity of nine chemotherapy drugs was analyzed and compared between the two risk groups in NSCLC. The results showed that the IC50 values of dasatinib, docetaxel, erlotinib, gemcitabine, paclitaxel, parthenolide, and shikonin were lower in the high-risk group than those in the low-risk group (*p* < 0.001, Figures 10A–G), whereas the IC50 values of axitinib and metformin were lower in the low-risk group than those in the high-risk group (*p* < 0.001, Figures 10H,I).

### Quantitative PCR validation

**FIGURE 11:**
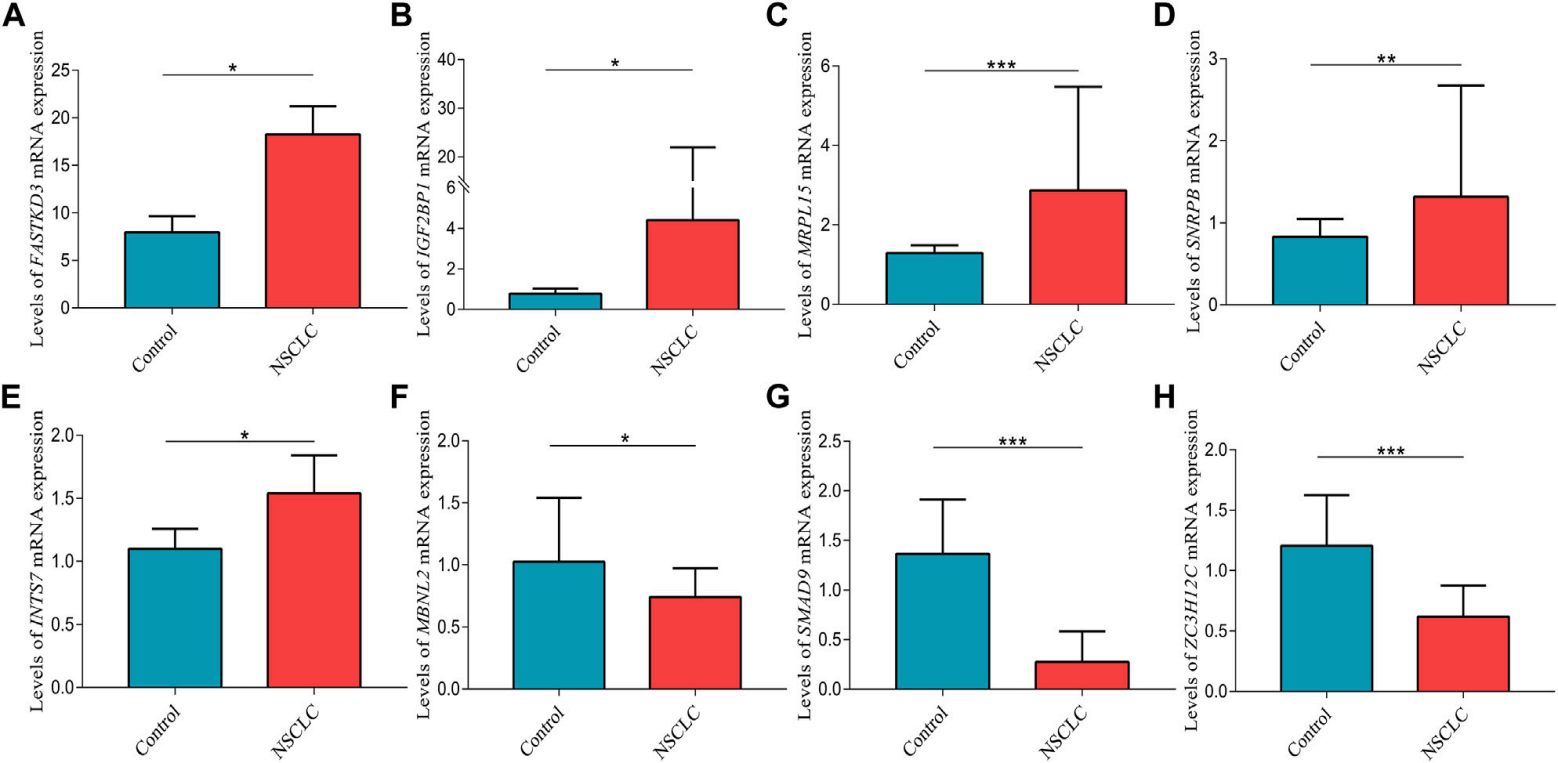
Validation of the relative expression levels of **(A)** *FASTKD3*, **(B)** *IGF2BP1*, **(C)** *MRPL15*, **(D)** *SNRPB*, **(E)** *INTS7*, **(F)** *MBNL2*, **(G)** *SMAD9*, and **(H)** *ZC3H12C* in lung tissues by qPCR. Data are presented as the median (interquartile range). NSCLC, non-small cell lung cancer; *p < 0.05; **p < 0.01; ***p < 0.001.

The mRNA expression levels of *FASTKD3* (*p* = 0.013), *IGF2BP1* (*p* = 0.026), *MRPL15* (*p* < 0.001), *SNRPB* (*p* = 0.005), and *INTS7* (*p* = 0.027) were higher in tumor tissues than in control tissues (Figures 11A–E); however, the mRNA expression levels of *MBNL2* (*p* = 0.015), *SMAD9* (*p* < 0.001), and *ZC3H12C* (*p* < 0.001) were decreased in tumor tissues compared to control tissues (Figures 11F–H).

## Discussion

In the present study, an RBP-related signature was successfully constructed to predict prognosis. The increased risk score was associated with more advanced tumor stages and lower OS probability. The risk score was an independent prognostic factor for NSCLC when adjusted for age and UICC stage. Moreover, the constructed nomogram better predicted prognosis. In addition, the high-risk group had increased immune infiltration, upregulated relative expression levels of *PD1* and *CTLA4*, higher TMB, and lower IC50 of chemotherapy drugs than the low-risk group.

KEGG pathway analysis was performed to explore the underlying molecular mechanisms of RBPs in NSCLC. Previous studies have demonstrated that the mRNA surveillance pathway is involved in the initiation and progression of cancer (Long et al., 2017; Popp and Maquat, 2018; Zhang et al., 2019a), which was confirmed in NSCLC in the present study. In addition, the present study showed that ribosome biogenesis in eukaryotes played an important role in NSCLC. RPL15, a large ribosomal subunit protein, is significantly upregulated in human cancer tissues and cultured cell lines, and it is closely correlated with clinicopathological characteristics (Dong et al., 2019). Interestingly, the present study demonstrated that influenza A was correlated with NSCLC. A previous study has reported that exposure to the influenza virus is associated with an increased risk of lung cancer and that the risk increases with cumulative exposure to influenza (Weng et al., 2019). Thus, the annual influenza vaccination administration may reduce the incidence of lung cancer in patients with COPD (Chen et al., 2019). However, the precise molecular mechanisms need to be further explored in future studies.

The molecular mechanisms through which these RBPs contribute to the pathogenesis of cancer remain poorly understood. *IGF2BP1* has been shown to function as an oncogene in multiple cancers (Ohdaira et al., 2012; Huang et al., 2020a; Glaß et al., 2020). Low *IGF2BP1* expression inhibits cell proliferation and migration but induces cell cycle arrest and apoptosis in NSCLC, and it correlates with a good prognosis (Huang et al., 2019; Huang et al., 2020b; Zhang et al., 2020). *MRPL15* is associated with the progression of LUAD (Deng et al., 2020). *SMAD9* is involved in the progression of NSCLC by miR-744 delivered by cancer-derived extracellular vehicles (Gao et al., 2021). Moreover, *SMAD9* mRNA expression is decreased in LUAD, which is correlated with worse OS (Dai et al., 2020). *SNRPB* promotes tumor cell proliferation and stemness by mediating RNA splicing (Zhan et al., 2020). In NSCLC, *SNRPB* facilitates tumorigenesis *via* regulation of *RAB26* expression, and it is correlated with prognosis (Liu et al., 2019). *MBNL2* inhibits tumor growth and metastasis (Lee et al., 2016; Zhang et al., 2019b). *MBNL2* controls lung cancer cell responses to hypoxia by regulating the expression and alternative splicing of hypoxia-induced genes (Fischer et al., 2020). However, there are few reports on *FASTKD3* and *ZC3H12C* involved in cancer. The results of the present study showed that the relative expression levels of *FASTKD3*, *IGF2BP1*, *MRPL15*, *SNRPB*, and *INTS7* were higher in NSCLC tissues than those in control tissues but that the relative expression levels of *MBNL2*, *SMAD9*, and *ZC3H12C* were reduced in NSCLC tissues compared to those in control tissues.

The prognostic RBP-related signatures have been constructed in LUAD (Meng et al., 2020; Yang et al., 2021) and LUSC (Li et al., 2020a; Zhao et al., 2021). However, the constructed RBP-related signatures for LUAD and LUSC only use simple bioinformatics analysis and lack experimental validation, and each signature is suitable for only one type of NSCLC. Thus, a prognostic RBP-related signature should be generated and validated for NSCLC. In the present study, a prognostic RBP-related signature was successfully constructed using LASSO regression analysis rather than multivariable Cox regression analysis based on eight RBPs that were validated using qPCR, which minimized the risk of overfitting among the signature and increased the reliability of the signature. Thus, the present signature was superior to the previously reported prognostic signatures for LUAD (Meng et al., 2020; Yang et al., 2021) and LUSC (Li et al., 2020a; Zhao et al., 2021). Kaplan–Meier survival analysis showed that the present signature predicted prognosis and discriminated against different risk groups. The risk score was positively correlated with tumor progression, including UICC stages, T stages, and N stages. However, there was no correlation between the risk score and M stages, which may have been due to an insufficient sample size in the M1 stage. Moreover, the risk score was an independent prognostic factor for NSCLC, according to multivariate Cox regression analysis. Thus, the risk score was associated with the progression and prognosis of NSCLC. Finally, the prognostic RBP-related signature was validated using four GEO datasets, which demonstrated that the prognostic signature was not restricted by different sequencing techniques and platforms. Previous studies have shown that a nomogram better predicts disease prognosis due to its multidimensional parameters (Huang et al., 2022; Luo et al., 2022). Thus, a nomogram was constructed in the present study to predict the 1-, 3-, and 5-year OS probability in NSCLC, and calibration plots of the nomogram showed high predictive accuracy.

The underlying molecular mechanisms between the two risk groups were investigated using GSEA. The pathways were mainly enriched in the *P53* signaling pathway and NOD-like receptor signaling pathway. As a canonical tumor suppressor, *P53* plays an important role in cancer (Yoshida and Miki, 2010; Timofeev et al., 2020). Mutant and wild-type *P53* may exert different functions on cancer (Yoshida and Miki, 2010; Muller and Vousden, 2014; Timofeev et al., 2020). *P53* is a frequently mutated gene in lung cancer (Oduah and Grossman, 2020), which was also confirmed using mutation analysis in the present study. The mutant *P53* promotes tumor progression by binding to and upregulating chromatin regulatory genes, such as *MLL1* and *MLL2*, leading to genome-wide increases in histone methylation and acetylation (Zhu et al., 2015). Moreover, the mutant *P53* accelerates the recycling of integrin beta1 and *EGFR* to exert its oncogenic function (Muller et al., 2009). Dysregulated TMB is correlated with the prognosis of cancer (Cao et al., 2022; Zhou and Gao, 2022). In the present study, the high-risk group had a higher TMB, and patients in the high-risk group with high TMB had a worse probability. In addition, activation of the NLR family pyrin domain containing 3 (*NLRP3*) inflammasome enhances the proliferation and migration of A549 cells (Wang et al., 2016). Tumor-derived exosomal *TRIM59* induces the tumor-promoting function of macrophages to activate the *NLRP3* inflammasome signaling pathway, thereby promoting lung cancer progression (Liang et al., 2020b). Thus, inhibiting *NLRP3* inflammasome activation may suppress cancer cell proliferation and metastasis in NSCLC (Zou et al., 2018). Nucleotide-binding and oligomerization domain-containing protein 2 (*NOD2*) deficiency confer a protumorigenic macrophage phenotype to promote LUAD progression (Wang et al., 2021). Therefore, *P53* mutation and the NOD-like receptor signaling pathway may play a critical role in disease progression and worse OS probability in the high-risk group.

Immune cells are an important part of the TME, and they play a critical role in tumor development (Bindea et al., 2013). In lung cancer, macrophages stimulate tumor angiogenesis and promote cancer cell invasion, migration, and intravasation (Qian and Pollard, 2010). Tumor-associated macrophages are significantly associated with angiogenesis and a poor prognosis in NSCLC (Montuenga and Pio, 2007; Li et al., 2020e). Neutrophils in peripheral blood are effective diagnostic biomarkers for lung cancer (Zhu et al., 2020). Increased neutrophils are associated with a worse prognosis in bronchoalveolar carcinoma (Bellocq et al., 1998). A high percentage of CD4^+^ tumor-infiltrating lymphocytes in the tumor stroma is correlated with a worse prognosis (Giatromanolaki et al., 2021). In the present study, increased M_0_ macrophages, M_1_ macrophages, neutrophils, and activated memory CD4^+^ T cells were found in the high-risk group. The worse OS probability in the high-risk group may be attributed to increased immune infiltration, indicating that the high-risk group may have a better immunotherapy response. Therefore, the correlations of risk scores with the relative expression levels of immune checkpoint inhibitors (*PD1*, *PDL1*, and *CTLA4*) were evaluated to further explore their association with immunotherapy because immune checkpoint inhibitors (ICI) are becoming standard in the first-line treatment of advanced NSCLC (Garon et al., 2015; Reck et al., 2016; Gandhi et al., 2018). The results showed that the high-risk group had higher relative expression levels of *PD1* and *CTLA4*, suggesting that patients in the high-risk group may benefit more from ICIs against *PD1* and *CTLA4*. In addition to immunotherapy, the correlations of risk scores with chemotherapeutic drugs were also explored. The results demonstrated that the high-risk group was more sensitive to seven chemotherapy drugs and that the low-risk group was more sensitive to two chemotherapy drugs. Thus, the prognostic signature may be applied to guide individualized chemotherapy choices.

The present study had several advantages. First, the signature was constructed using LASSO regression analysis, which minimized the risk of overfitting among the signature. Second, the present study established a promising prognostic RBP-related signature to evaluate patient prognosis, and we performed comprehensive bioinformatics analysis, including correlation of the risk score with clinical characteristics, methylation levels, TMB, copy number variation, immune infiltration, and chemotherapy response, as well as GSEA between the two risk groups, which have not been performed in previous RBP-related signatures for LUAD (Meng et al., 2020; Yang et al., 2021) and LUSC (Li et al., 2020a; Zhao et al., 2021). Third, the previously constructed RBP-related signatures lack experimental validation, but the dysregulated genes in the present prognostic signature were validated using qPCR in another independent cohort. Fourth, the prognostic RBP-related signature was validated using four GEO databases. Last, the present RBP-related signature was generated for all NSCLC cohorts rather than one subgroup of NSCLC. Nevertheless, the present study had several limitations. First, the results of the present study were based on bioinformatics analyses of public databases, which need to be validated in multicentric, prospective clinical studies. Second, Kaplan–Meier survival curve analysis could not be performed using our samples due to insufficient sample size and lack of follow-up data. Last, the present study did not perform *in vivo* and vitro experiments to explore the function of RBPs and prognostic signatures. Thus, further studies are required to clarify the molecular mechanism of RBPs in NSCLC.

## Conclusion

A prognostic RBP-related signature was successfully constructed based on eight RBPs using LASSO regression analysis. The risk score was associated with progression of disease and OS probability, and it was an independent prognostic factor for NSCLC. Moreover, the high-risk group had increased immune infiltration, upregulated relative expression levels of *PD1* and *CTLA4*, higher gene mutation frequency, higher TMB, and better chemotherapy response. Therefore, an RBP-related signature was successfully constructed to predict prognosis in NSCLC, which may function as a reference for individualized therapy, including immunotherapy and chemotherapy.

## Data Availability

The original contributions presented in the study are included in the article/Supplementary Material; further inquiries can be directed to the corresponding author.
